# Supplementary figures and images for: A case of long-term herbivory: specialized feeding trace on Parrotia (Hamamelidaceae) plant species
Source: R Soc Open Sci. 2020 Oct 28;7(10):201449. doi: 10.1098/rsos.201449 (PMC7657907; doi:10.1098/rsos.201449)

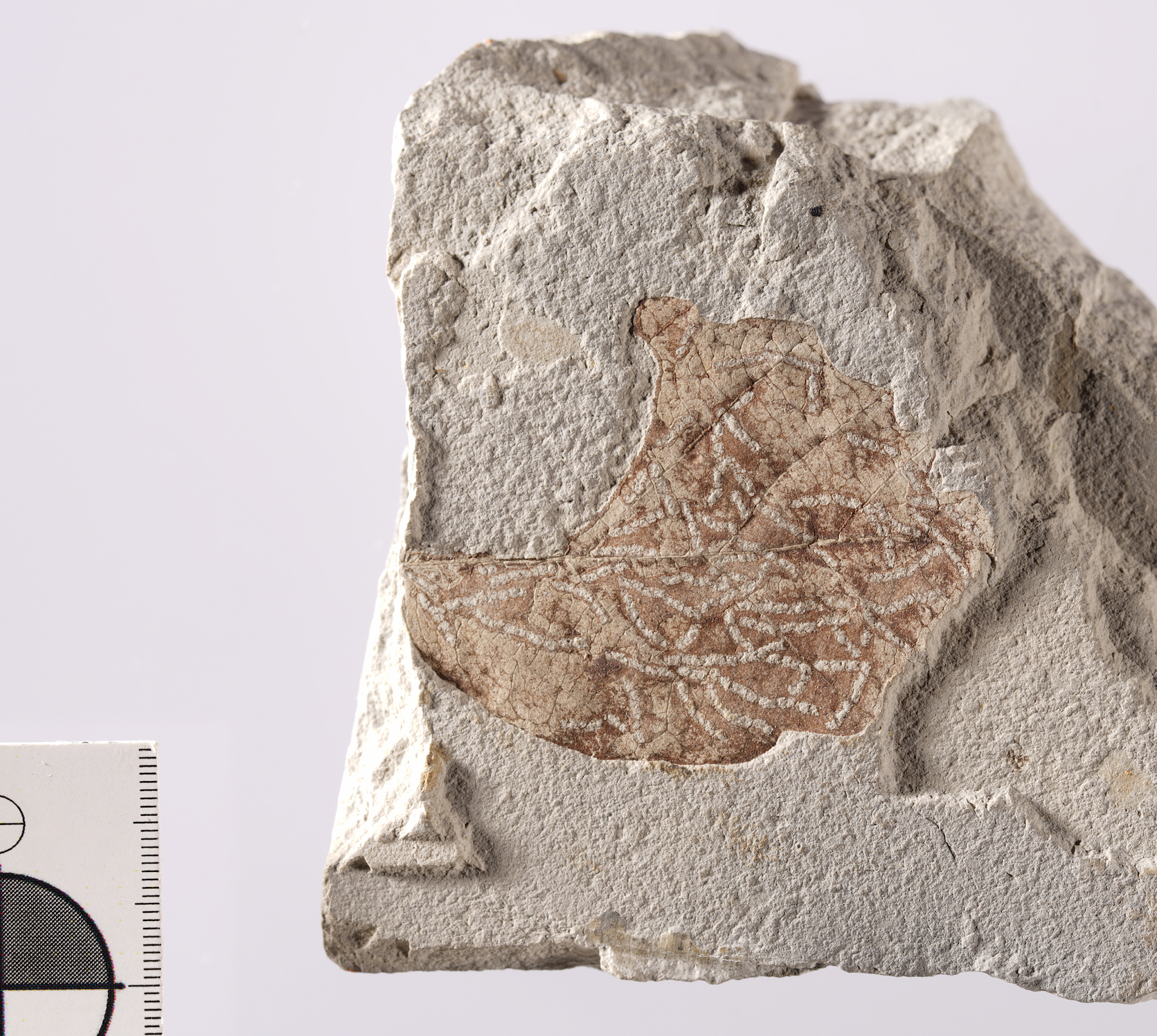

Supplement: Supplement 1: Parrotia persica with DT297 occurrences. [file rsos201449supp1.tiff]

# Leaf Mass per Area

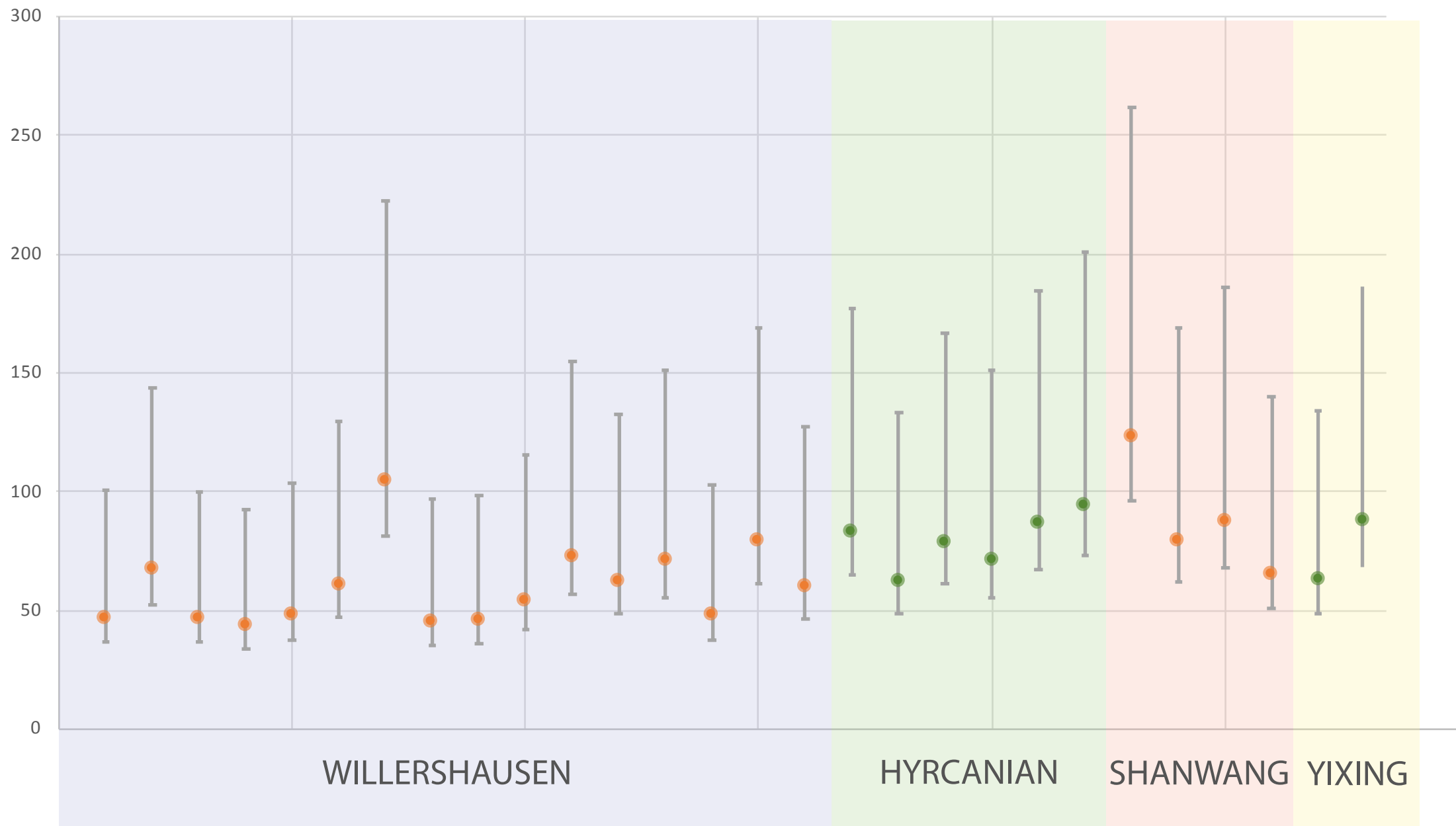

Supplement: Supplement 2: Leaf mass per area for every single leaf which include DT297 on their blade [file rsos201449supp2.pdf]
